# Levocarnitine for pegaspargase‐induced hepatotoxicity in older children and young adults with acute lymphoblastic leukemia

**DOI:** 10.1002/cam4.4281

**Published:** 2021-09-16

**Authors:** Rachael Schulte, Ashley Hinson, Van Huynh, Erin H. Breese, Joanna Pierro, Seth Rotz, Benjamin A. Mixon, Jennifer L. McNeer, Michael J. Burke, Etan Orgel

**Affiliations:** ^1^ Division of Pediatric Hematology and Oncology Vanderbilt University Medical Center Nashville Tennessee USA; ^2^ Division of Pediatric Hematology and Oncology Levine Children's Atrium Health Charlotte North Carolina USA; ^3^ Division of Pediatric Oncology University of California Irvine College of Medicine CHOC Children's Hospital Orange California USA; ^4^ Division of Oncology, Cancer and Blood Diseases Institute Cincinnati Children's Hospital Medical Center/University of Cincinnati College of Medicine Cincinnati Ohio USA; ^5^ Division of Pediatric Hematology/Oncology NYU Grossman School of Medicine Perlmutter Cancer Center Hassenfeld Children's Hospital at NYU Langone Health New York New York USA; ^6^ Department of Pediatric Hematology, Oncology, and Blood and Marrow Transplantation Pediatric Institute Cleveland Clinic Foundation Cleveland Ohio USA; ^7^ Department of Pediatrics University of Tennessee College of Medicine Chattanooga and Children's Hospital at Erlanger Chattanooga Tennessee USA; ^8^ Section of Pediatric Hematology, Oncology, and Stem Cell Transplant University of Chicago Comer Children's Hospital Chicago Illinois USA; ^9^ Division of Pediatric Hematology/Oncology and Blood and Marrow Transplantation Medical College of Wisconsin Milwaukee Wisconsin USA; ^10^ Cancer and Blood Disease Institute Children's Hospital Los Angeles/University of Southern California Los Angeles California USA; ^11^ Present address: Center for Cancer and Blood Disorders Riley Children's Hospital/Indiana University School of Medicine Indianapolis Indiana USA

**Keywords:** adolescent, asparaginase, carnitine, chemical and drug‐induced liver injury, precursor cell lymphoblastic leukemia‐lymphoma, young adult

## Abstract

**Background:**

Pegaspargase (PEG‐ASP) is an integral component of therapy for acute lymphoblastic leukemia (ALL) but is associated with hepatotoxicity that may delay or limit future therapy. Obese and adolescent and young adult (AYA) patients are at high risk. Levocarnitine has been described as potentially beneficial for the treatment or prevention of PEG‐ASP‐associated hepatotoxicity.

**Methods:**

We collected data for patients age ≥10 years who received levocarnitine during induction therapy for ALL, compared to a similar patient cohort who did not receive levocarnitine. The primary endpoint was conjugated bilirubin (c.bili) >3 mg/dl. Secondary endpoints were transaminases >10× the upper limit of normal and any Grade ≥3 hepatotoxicity.

**Results:**

Fifty‐two patients received levocarnitine for prophylaxis (*n* = 29) or rescue (*n* = 32) of hepatotoxicity. Compared to 109 patients without levocarnitine, more patients receiving levocarnitine were obese and/or older and had significantly higher values for some hepatotoxicity markers at diagnosis and after PEG‐ASP. Levocarnitine regimens varied widely; no adverse effects of levocarnitine were identified. Obesity and AYA status were associated with an increased risk of conjugated hyperbilirubinemia and severe transaminitis. Multivariable analysis identified a protective effect of levocarnitine on the development of c.bili >3 mg/dl (OR 0.12, *p *= 0.029). There was no difference between groups in CTCAE Grade ≥3 hepatotoxicity. C.bili >3 mg/dl during induction was associated with lower event‐free survival.

**Conclusions:**

This real‐world data on levocarnitine supplementation during ALL induction highlights the risk of PEG‐ASP‐associated hepatotoxicity in obese and AYA patients, and hepatotoxicity's potential impact on survival. Levocarnitine supplementation may be protective, but prospective studies are needed to confirm these findings.

## Graphical abstract

Patients who are adolescents or young adults and/or who are obese are at higher risk for pegaspargase‐induced hepatotoxicity. Levocarnitine supplementation may reduce the risk for hepatoxicity during induction therapy for acute lymphoblastic leukemia.

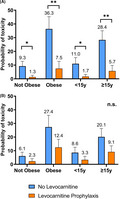

## 1 INTRODUCTION

Incorporation of asparaginase into treatment for acute lymphoblastic leukemia (ALL) has reduced relapse rates and improved survival for children.^1^ Recently, adoption of pediatric‐inspired regimens inclusive of asparaginase has similarly improved survival for adolescent and young adult (AYA) patients with ALL.^2^, ^3^ Unfortunately, the use of asparaginase‐containing regimens in AYA patients is complicated by hepatotoxicity in up to 60% of patients,^3^, ^4^, ^5^, ^6^, ^7^ with obesity conferring additional increased risk.^3^, ^4^, ^5^, ^7^, ^8^, ^9^, ^10^, ^11^ Histopathology of affected livers shows fatty infiltration lasting weeks to months^12^ with severity ranging from mild elevations of bilirubin and/or transaminases to fulminant liver failure.^6^, ^7^ Asparaginase‐induced hepatotoxicity results in significant morbidity, omission or dose reductions of chemotherapy, and in some cases, mortality.^9^ Thus, hepatotoxicity complicates the use of asparaginase in contemporary treatment regimens, particularly for vulnerable obese and/or AYA patients.

Traditional strategies to facilitate safe incorporation of asparaginase have focused on pharmacodynamic dosing intended to reduce drug exposure (e.g., reduced dose or dose‐capping),^13^, ^14^ but an emerging emphasis on hepatoprotective agents may offer alternative and/or complementary approaches.^7^, ^15^ Within the liver, endogenous carnitine contributes to energy needs via fatty acid delivery and oxidation within the mitochondria and by buffering excess organic acids to maintain cellular viability. Levocarnitine and its analogues have demonstrated benefit to mitigate damage from other forms of drug‐induced hepatotoxicity.^16^, ^17^ Preclinical data and isolated case reports have led to increased off‐label use of levocarnitine supplementation to potentially limit asparaginase‐induced hepatotoxicity,^18^, ^19^, ^20^, ^21^, ^22^, ^23^, ^24^, ^25^ including recommendations for its use in AYA populations.^15^ However, there is a paucity of clinical data demonstrating safety and efficacy of incorporating levocarnitine supplementation into ALL therapy. As a first step in addressing this critical knowledge gap, we conducted a study to investigate real‐world data on the safety and efficacy of levocarnitine supplementation during ALL induction therapy.

## 2 METHODS

Demographic, diagnosis, and treatment information were collected on patients treated with a Children's Oncology Group (COG)‐style ALL induction regimen inclusive of pegylated l‐asparaginase (PEG‐ASP) (Table S1). No patients received other formulations of asparaginase during induction therapy. All patients with newly‐diagnosed ALL who received levocarnitine during induction therapy were identified independently at each center and included for data capture (from 10 centers, treated 2014–2019). The indication for supplementation with levocarnitine, dosing regimens, and stopping criteria were determined by the treating clinicians and not standardized. Off‐label use of levocarnitine for prophylaxis was nearly uniformly prescribed by providers to patients ≥10 years old, with analyses therefore limited to this group (one outlier patient <10 years old receiving levocarnitine prophylaxis for an anthracycline‐sparing induction regimen was therefore excluded from analyses). This age cutoff is also in alignment with a recent study of risk factors for hepatotoxicity during ALL induction therapy.^26^ A second cohort was assembled from unselected and consecutively treated ALL patients ≥10 years old treated with the same COG induction chemotherapy without levocarnitine (from two centers, treated 2009–2019) (Table 1). Study endpoints were side effects from levocarnitine supplementation, incidence of hepatotoxicity, and disease response (minimal residual disease at end of induction [EOI MRD], event‐free survival [EFS], and overall survival [OS]).

**TABLE 1:** Description of cohort

Variable	Levocarnitine prophylaxis		Levocarnitine rescue		No levocarnitine		*p*‐value	
	N	(%)	N	(%)	N	(%)	All Groups	Prophylaxis versus no levocarnitine
Cohort	29	(100)	23	(100)	109	(100)		
Age, years								
10–14.9	8	(28)	3	(13)	57	(52)	0.001	0.018
15.0–39.9 (AYA)	21	(72)	20	(87)	52	(48)		
Median (range)	17.0	(12–26.3)	18.8	(11.7–35)	14.7	(10–27.2)	<0.001	0.001
Sex								
Female	8	(28)	6	(26)	42	(39)	0.349	0.276
Male	21	(72)	17	(74)	67	(61)		
Ethnicity								
Not Hispanic/Latinx	21	(72)	13	(56)	29	(27)	<0.001	<0.001
Hispanic/Latinx	4	(14)	10	(44)	70	(64)		
Unknown	4	(14)	0	(0)	10	(9)		
BMI category^a^								
Not obese	6	(21)	10	(43)	76	(70)	<0.001	<0.001
Obese	23	(79)	13	(57)	33	(30)		
BMI percentile								
Median (range)	98.3	(18.1–99.8)	98.8	(19.3–99.7)	79.0	(0.2–99.8)	0.001	0.001
Diagnosis								
B‐ALL	28	(97)	21	(91)	89	(82)	0.105	0.076
T‐ALL	1	(3)	2	(9)	20	(18)		
Presenting WBC^b^								
<50 K/ul	17	(59)	13	(56)	76	(70)	0.280	0.229
≥50 K/ul	12	(41)	10	(44)	32	(30)		
FISH/Cytogenetics^c^								
Neutral	11	(39)	9	(43)	37	(42)	0.002	0.004
Favorable	1	(4)	0	(0)	9	(10)		
Adverse	16	(57)	12	(57)	25	(28)		
Unknown	0	(0)	0	(0)	18	(20)		

Abbreviations: ALL, acute lymphoblastic leukemia; BMI, body mass index; FISH, Fluorescence in situ hybridization; WBC, white blood cell count.

^a^ Obesity categorized using age/sex norms for age 10–20 years and absolute BMI ≥30 for age ≥20 years (absolute BMI not included in median/range).

^b^ One patient unknown.

^c^ For B‐ALL patients, classified using Children's Oncology Group biology protocol AALL08B1 (modified to include Ph‐like signatures as adverse prognostic markers).

The primary hepatotoxicity endpoint was defined as conjugated bilirubin (c.bili) >3 mg/dl. This is the threshold in contemporary ALL protocols for dose modification of the hepatically metabolized induction chemotherapy agents (daunorubicin, vincristine). As minor elevations in aspartate (AST) and/or alanine aminotransferase (ALT) are common in ALL therapy, a secondary endpoint assessed severe transaminitis (defined for this study as >10× the upper limit of normal (ULN); ULN for AST and ALT were set at 50 and 45 U/L, respectively). To enable inter‐study comparisons, elevations in bilirubin and/or transaminitis were also graded using Common Terminology Criteria for Adverse Events (CTCAE) v4.03 to describe Grade ≥3 hepatotoxicity. MRD was quantified by flow cytometry at COG‐certified laboratories (MRD positive cutoff set at ≥0.01%)^27^; this endpoint was limited to patients with B‐ALL. MRD was assessed at the end of the induction phase (EOI), and in those with EOI MRD ≥0.01%, at the end of the consolidation phase (EOC) following an additional ~8 weeks of cytotoxic chemotherapy. EFS was defined as time from diagnosis to last follow‐up or first relapse, second malignancy, or death, and OS as time from diagnosis to death.

Levocarnitine supplementation was defined as “prophylaxis” when started anytime prior to PEG‐ASP exposure.^28^ As the rationale for starting supplementation after PEG‐ASP was not generally known (and was potentially due to early evidence or concerns for hepatotoxicity), levocarnitine started anytime following the first dose of PEG‐ASP was classified as “rescue.” AYA age was classified as 15–39 years old at diagnosis.^29^

### 2.1 Statistical methods

Based on recent data for ALL induction hepatotoxicity,^5^, ^10^, ^30^ multivariable models for each hepatotoxicity endpoint were constructed using a preselected model inclusive of age, obesity, ethnicity, number of PEG‐ASP doses in induction (1 or 2), and levocarnitine prophylaxis. Influence of sex was tested against this model using the likelihood ratio test (retained for *p *< 0.15). For clinical relevance, odds ratios were translated into the predicted probability for each hepatotoxicity endpoint and average marginal effects (AME)^31^ from incorporating levocarnitine prophylaxis were calculated, stratified by obesity and AYA age. A secondary analysis followed the same approach and examined levocarnitine supplementation started anytime after initiation of chemotherapy and prior to the onset of hepatotoxicity (c.bili >3 mg/dl). Logistic regression models for EOI MRD were constructed with prognostic covariables of presenting white blood cell count (WBC), age, obesity, and cytogenetic category. Impact of c.bili >3 mg/dl during induction and ethnicity was tested against the model (retained for *p* < 0.15). Cox multivariable models were constructed for EFS and OS using the same approach. Levocarnitine exposure (any) was then tested against each model for a potential association with MRD or survival.^32^ All statistical tests were two‐sided and significance set at *p* < 0.05. Calculations were performed using STATA Statistical Software, SE Release 15.0 (StataCorp, LLC).

## 3 RESULTS

Data were collected on a total of 161 patients, 52 of whom received levocarnitine for prophylaxis (29/52 [56%]) or rescue (23/52 [44%]) during induction therapy. Of those patients receiving levocarnitine for rescue following administration of PEG‐ASP, 10/23 (44%) began supplementation following elevated c.bili >3 mg/dl. Data for comparison were collected from 109 patients receiving induction chemotherapy without levocarnitine exposure. As shown in Table 1, patients receiving levocarnitine for prophylaxis were significantly more obese and older than patients without levocarnitine prophylaxis. Baseline hepatic function at diagnosis were more likely to show significant abnormalities from leukemic infiltration in those receiving levocarnitine supplementation (Table 2). Most patients received a single dose of PEG‐ASP during induction therapy (154/161 [96%]) with several receiving two doses (7/161 [4%]). The PEG‐ASP dose of 2500 IU/m^2^ was “capped” at 3750 IU for 4% (6/161) of patients; the administered dose of PEG‐ASP exceeded 3750 IU in the majority of patients (125/161 [77%]).

**TABLE 2:** Selected laboratory values for each cohort

	No levocarnitine	Levocarnitine prophylaxis	*p*‐value^a^	Levocarnitine rescue	*p*‐value^a^
At diagnosis					
AST, U/L median (range)	39 (13–901)	56 (15–708)	0.109	53 (16–188)	0.353
ALT, U/L median (range)	33 (6–840)	86 (13–1,257)	<0.001	81 (6–314)	0.005
T.bili, mg/dl median (range)	0.6 (0.1–4.4)	0.8 (0.3–11.8)	0.009	0.6 (0.2–2.8)	0.249
C.bili, mg/dl median, range)	0.4 (0.0–2.2)	0.5 (0.0–7.3)	0.335	0.2 (0.0–1.1)	0.095
Peak After PEG‐ASP					
AST, U/L median (range)	63 (11–6,789)	89 (16–1,493)	0.166	181 (34–6,297)	<0.001
ALT, U/L median (range)	140 (32–2850)	258 (20–862)	0.031	431 (112–2160)	<0.001
T.bili, mg/dl median (range)	1.5 (0.3–26.7)	2.4 (0.5–20.8)	0.015	6.4 (1.2–26.3)	<0.001
C.bili, mg/dl median, range	0.7 (0.0–20.7)	1.1 (0.0–19.9)	0.049	3.3 (0.0–21.8)	0.001

^a^ Significance in comparison to no levocarnitine cohort.

### 3.1 Levocarnitine supplementation

In those receiving levocarnitine prophylaxis, levocarnitine was started at a median of three days (range 1–30 days) prior to the first PEG‐ASP dose, including one patient who received prolonged supplementation prior to PEG‐ASP during treatment prophase. In the levocarnitine rescue group, levocarnitine was started a median of 18 days (range 0–43) following PEG‐ASP, and, in the subset who developed c.bili >3 mg/dl, a median of 2 days (range 0–25) after c.bili exceeded >3 mg/dl. Levocarnitine dosing regimens varied widely, ranging from 330 to 1980 mg/dose PO or IV administered every 4–12 h (dose range 660–6400 mg/day); the most common dose was ~1000 mg TID (3000 mg/day) in 33/52 (64%). There was no significant difference in the mean daily dose delivered for prophylaxis vs rescue (2664 mg [SD 712] vs. 3,102 mg [SD 1387], *p *= 0.147). Levocarnitine supplementation (prophylaxis or rescue) was administered for a total of 4053 days (median 29 days/patient, range 1–872 days; one site treated for the duration of therapy). No side effects or early discontinuation were reported with levocarnitine supplementation.

### 3.2 Prevention of hepatotoxicity

In the overall cohort, 34/161 (21%) developed c.bili >3 mg/dl, 27/161 (17%) developed study‐defined severe transaminitis, and 16/161 (10%) developed both. CTCAE Grade ≥3 hepatotoxicity (transaminitis or hyperbilirubinemia) was reported in 83/161 (52%) of patients. More patients with obesity developed c.bili >3 mg/dl than those non‐obese (21/69 [30%] vs. 13/92 [14%], *p *= 0.012) as did more AYA patients versus those younger (26/93 [28%] vs. 8/68 [12%], *p *= 0.013). Among those with known ethnicity, the rate of c.bili >3* *mg/dl was similar in those with self‐reported Hispanic/Latinx ethnicity versus non‐Hispanic/Latinx (16/84 [19%] vs. 25/63 [24%], *p *= 0.484). Similar trends were present for the secondary endpoint of severe transaminitis (obese vs. non‐obese 17/69 [25%] vs. 10/92 [11%], *p *= 0.021; AYA vs. non‐AYA 21/93 [23%] vs. 6/68 [9%], *p* = 0.031).

**FIGURE 1:**
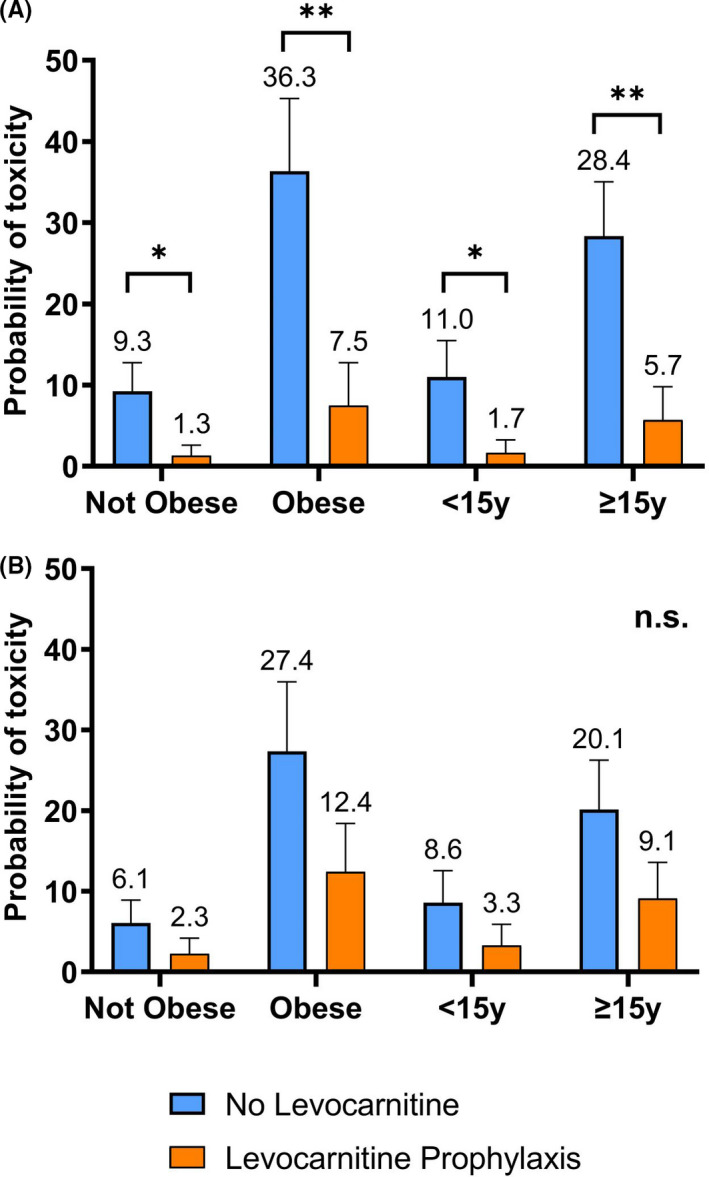
Probability of developing hepatotoxicity following PEG‐ASP exposure. Multivariable models were constructed for each hepatotoxicity endpoint. Predicted probability of developing (A) conjugated bilirubinemia >3 mg/dl or (B) severe transaminitis (defined as aspartate or alanine aminotransferase >10× upper limit of normal) were calculated for patients with or without levocarnitine prophylaxis and stratified by at‐risk populations (obesity, adolescent & young adult). *p < 0.05, **p < 0.01, n.s., not significant

**FIGURE 2:**
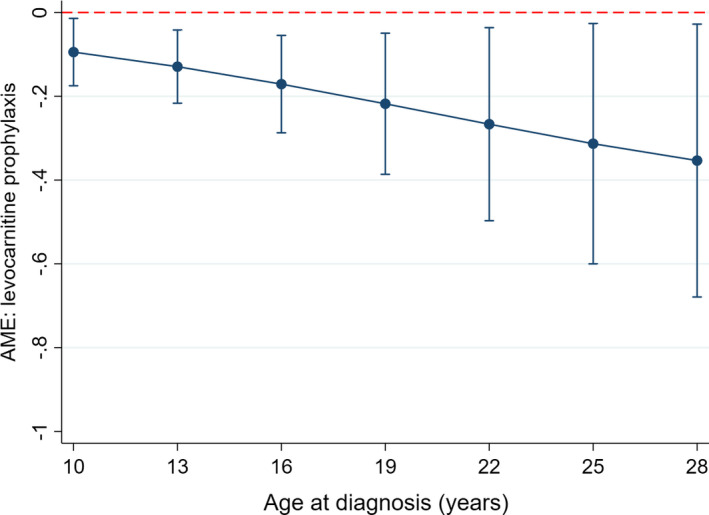
Average marginal effects from the incorporation of levocarnitine prophylaxis on the probability of conjugated bilirubin >3 mg/dl. From the multivariable logistic regression model for the endpoint of conjugated bilirubin >3 mg/dl, average marginal effects (AME) were calculated with associated 95% confidence intervals for the incorporation of levocarnitine prophylaxis

There was no significant difference in overall prevalence of c.bili >3* *mg/dl or severe transaminitis in those receiving prophylaxis versus no prophylaxis (14% [4/29] vs. 16% [17/109], *p *= 0.810 and 21% [6/29] vs. 10% [11/109], *p *= 0.123). However, multivariable analysis accounting for the above at‐risk populations demonstrated a significant protective effect of levocarnitine prophylaxis on risk for developing dose‐limiting c.bili >3 mg/dl (odds ratio [OR] 0.12, 95% confidence interval [95% CI] 0.02–0.81, *p* = 0.029) but not for severe transaminitis (OR 0.34, 95% CI 0.07–1.73, *p* = 0.194) (Table 3). Analysis of AME showed incorporating levocarnitine prophylaxis into COG‐style ALL induction therapy was predicted to significantly reduce the probability of developing c.bili >3 mg/dl (dy/dx −16.5%, 95% CI −0.27 to −0.06, *p *= 0.002) but not severe transaminitis (dy/dx −0.08%, 95% CI −0.19 to +0.03, *p *= 0.139). The predicted probability for each endpoint within high‐risk population subsets (obesity, AYA) is shown in Figure 1. A trend was present for greater benefit from levocarnitine supplementation in those with older age at diagnosis (Figure 2). Secondary analyses reclassifying levocarnitine supplementation only as prior to or post‐onset of hepatotoxicity irrespective of PEG‐ASP timing showed similar trends (Table S2). Levocarnitine supplementation did not alter the risk for CTCAE grade ≥3 hepatotoxicity (OR 1.14, 95% CI 0.38–3.47, *p *= 0.812) (Table S3).

**TABLE 3:** Multivariable analysis of levocarnitine prophylaxis and hepatotoxicity endpoints

Covariable	Conjugated bilirubin >3 mg/dl			AST or ALT >10× ULN		
	OR	95% CI	*p*‐value^a^	OR	95% CI	*p*‐value^a^
Age						
<15 years	Reference			Reference		
≥15 years	4.27	1.22–14.93	0.023	3.77	0.95–14.96	0.059
BMI Category^b^						
Not obese	Reference			Reference		
Obese	6.14	1.88–20.05	0.003	5.02	1.25–20.18	0.023
Ethnicity^c^						
Not hispanic/Latinx	Reference			Reference		
Hispanic/Latinx	0.72	0.21–2.51	0.603	0.27	0.07–1.10	0.069
PEG‐ASP, # doses						
One	Reference			Reference		
Two	2.65	0.24–29.41	0.427	2.62	0.24–28.90	0.431
Levocarnitine suppl.						
No levocarnitine	Reference			Reference		
Prophylaxis	0.13	0.02–0.81	0.029	0.34	0.07–1.73	0.194

Abbreviations: 95% CI, 95% Confidence IntervalBMI, body mass index; PEG‐ASP, pegylated asparaginase; ULN, upper limit of normal.

^a^ Logistic regression model, see methods; biologic sex was not significant in either model (*p *= 0.629 & *p *= 0.882, respectively).

^b^ Obesity defined according to population norms for BMI in patients <20 years old (BMI percentile <95% vs. ≥95% and BMI <30 vs. ≥30 in patients ≥20 years old).

^c^ Restricted to those with known ethnicity.

### 3.3 Treatment of hepatotoxicity

In patients who developed dose‐limiting c.bili >3 mg/dl and received levocarnitine rescue following PEG‐ASP exposure versus no levocarnitine supplementation (n=10 vs. *n* = 15), there was no significant difference in mean days to the start of the next treatment phase for those who survived induction (42.5 days [SD 9.9] vs. 40.3 days [SD 9.1], *p *= 0.573). Duration from c.bili >3* *mg/dl until resolution <3* *mg/dl was significantly longer in those selected to receive rescue versus no supplementation (12 days [IQR 24] vs. 3 days [IQR 12], *p *= 0.026). In the 8/10 patients starting levocarnitine only after c.bili >3* *mg/dl, and who recovered from the toxicity, the median duration to resolution was 16.5 days (IQR 21). Induction deaths were rare (9/161 [6%]); among those who developed c.bili >3* *mg/dl, there was no difference in the rate of induction deaths in those who received rescue versus no supplementation (3/13 [23%] vs. 3/17 [18%], *p *= 0.713). Peak levels of bilirubin and transaminases were significantly higher in the rescue cohort as compared to those without levocarnitine exposure (Table 2).

### 3.4 ALL disease response and survival

In the subset of 115 patients with B‐ALL, known cytogenetics, and known end of induction (EOI) minimal residual disease (MRD), 47/115 (41%) received exogenous levocarnitine (prophylaxis 27/47 [57%], rescue 20/47 [43%]) and 68/115 (59%) did not. A higher prevalence of adverse cytogenetic features and fewer favorable genomic findings was present in levocarnitine supplemented patients (Table 1). MRD ≥* *0.01% was present at EOI in 44/115 (38%) and at EOC in 13/105 (12%). Levocarnitine supplementation was not associated with risk of MRD ≥0.01% (OR 1.68, 95% CI 0.62–4.58, *p *= 0.309) (Table 4). Analysis for the EOC MRD timepoint was limited by the lower prevalence of the persistent MRD positivity, but a multivariable analysis model inclusive only of cytogenetic risk and levocarnitine supplementation showed persistent influence of adverse cytogenetics (OR 5.2, 95% CI 1.29–20.63, *p *= 0.020) but no difference from levocarnitine supplementation (OR 0.68, 95% CI 0.19–2.37, *p *= 0.541). At a mean follow‐up of 2.4 years (SD 2.1) for surviving patients, EFS and OS showed a significant difference for patients who developed c.bili >3* *mg/dl during induction versus those who did not (3‐year EFS 40 ± 11/.2% vs. 77 ± 5.3%, *p *< 0.001; 3‐year OS 58 ± 9.7% vs. 88 ± 4.0%, *p *< 0.001) (Figure SA,B). In multivariable analysis, incorporation of levocarnitine did not impact EFS or OS (Table S4).

**TABLE 4:** Multivariable analysis of levocarnitine supplementation and EOI MRD ≥0.01%

Covariable	OR	95% CI	*p*‐value^a^
Age, years			
<15 years	Reference		
≥15 years	1.12	0.49–2.54	0.534
BMI Category^b^			
Not obese	Reference		
Obese	1.67	0.74–3.77	0.215
Presenting WBC			
<50 K/uL	Reference		
≥50 K/uL	3.32	1.42–7.72	0.005
FISH/Cytogenetics^c^			
Neutral	Reference		
Favorable	0.41	0.05–3.68	0.424
Adverse	2.40	0.98–5.89	0.056
Unknown	1.32	0.36–4.82	0.677
Levocarnitine suppl.^a^	1.68	0.62–4.58	0.309

Abbreviations: BMI, body mass index; FISH, Fluorescence in situ hybridization; WBC, white blood cell count.

^a^ Multivariable logistic regression model constructed from prognostic factors and levocarnitine supplementation then tested against model, see methods.

^b^ Obesity defined according to population norms for BMI in patients <20 years old (BMI percentile <95% vs. ≥95% and BMI <30 vs. ≥30 in patients ≥20 years old).

^c^ Classified as per Children's Oncology Group biology protocol AALL08B1 (modified to include Ph‐like signatures as adverse prognostic markers).

## 4 DISCUSSION

This study highlights that patients who are obese and/or AYA constitute vulnerable populations at significantly higher risk for dose‐limiting hepatotoxicity following treatment with PEG‐ASP. Though acute mortality from hepatotoxicity is rare, our findings also demonstrate a significant impact on survival from dose‐limiting toxicity during induction therapy. To address these challenges, this is the first multicenter study to evaluate off‐label usage of levocarnitine supplementation to prevent and/or rescue PEG‐ASP‐induced hepatotoxicity. Incorporation of levocarnitine was well tolerated with no associated adverse events reported in any patient and no indication of an adverse interaction with chemotherapy on disease response and survival. Despite the lack of a standardized approach to the incorporation of levocarnitine, a clear efficacy signal from levocarnitine prophylaxis was evident in patients at high risk for hepatotoxicity who received levocarnitine prophylaxis prior to PEG‐ASP. Patients in this group had significantly higher values for ALT and total bilirubin prior to the start of therapy and higher peak values for ALT, total bilirubin, and c.bili following PEG‐ASP (Table 2), likely reflecting their overall greater risk. However, in multivariable analysis accounting for these risk factors, levocarnitine prophylaxis reduced the odds of developing conjugated hyperbilirubinemia (Table 3). While this retrospective data is encouraging that levocarnitine may reduce rates of severe hepatotoxicity, the importance of a randomized controlled trial to validate the efficacy of levocarnitine prophylaxis cannot be understated.

Not surprisingly from use of an off‐label medication, data from this study also highlights the wide variety in dosing regimens, starting/stopping criteria, and indications for levocarnitine supplementation in patients with ALL. Optimal dosing has yet to be determined. Most patients in this study were treated using 50–100 mg/kg/day, divided into two or three doses, with a maximum dose of 3 g/day, as previously recommended,^18^ and most patients were treated with oral supplementation. This regimen remains reasonable as intestinal absorption of levocarnitine is generally saturated at 1 gram/dose, repeated daily dosing of ≥2 grams/day increases total body carnitine, and with only ~1% of total body carnitine present in the liver, prolonged exposure is likely required to maximize hepatic concentrations.^33^ However, it is unknown if lower or less frequent dosing retains benefit, and conversely, whether higher dosing may be necessary in some situations.

Contrary to earlier anecdotal evidence,^18^, ^20^, ^21^, ^24^ no clear benefit was found for levocarnitine rescue following severe hepatotoxicity. Patients developing severe hepatotoxicity did not rapidly recover and the duration of toxicity was similar to published non‐supplemented cohorts.^8^, ^10^ It is unknown if higher doses of levocarnitine or, alternatively, intravenous dosing to maximize delivery to the liver may have offered additional benefit. Thus, as opposed to the potential benefit from levocarnitine to prevent hepatotoxicity, no clear efficacy signal was present for levocarnitine rescue. A prospective trial will help further explore these continued knowledge gaps.

Understanding possible etiologies for susceptibility to hepatotoxicity, and particularly the contribution of pre‐existing non‐alcoholic fatty liver disease (NAFLD),^34^ will help to better guide levocarnitine use. Patients with NAFLD have altered mitochondrial respiratory chain function, including perturbations in carnitine shuttling.^35^ Oxidant stress from PEG‐ASP exposure might exacerbate mitochondrial stress from NAFLD, resulting in progression to non‐alcoholic steatohepatitis. This has been demonstrated in mice, where obesity causes activation of maladaptive pathways in hepatocytes in response to asparaginase‐induced metabolic stress, leading to reduced ability of the cells to be rescued from this stress by normal pathways.^36^ NAFLD is common in the AYA population, affecting 10% of non‐obese and 25%–50% of obese patients.^37^, ^38^ Understanding the prevalence and impact of NAFLD on PEG‐ASP toxicity and the potential role for levocarnitine supplementation is critical to reducing treatment morbidity from ALL induction therapy.

There are several limitations innate to this type of study. Given the off‐label usage of levocarnitine, there was no standardized supplementation regimen. There was also likely selection bias in determining which patients were treated with levocarnitine as either prophylaxis or rescue. However, we would note this bias further supports evidence for efficacy since a benefit was seen even in those patients perceived to be at highest risk of hepatotoxicity. Older patients are at the higher risk for hyperbilirubinemia and transaminitis from induction therapy than younger patients^11^; thus, the benefits of levocarnitine may be even greater in an older AYA population. Conversely, data in our cohort for those <15 years of age was sparse but statistical modeling supported potential efficacy in this group. If levocarnitine proves effective in randomized trials in AYA patients, its role in preventing hepatotoxicity for younger at‐risk patients should be explored. Moreover, the observed effect size from levocarnitine prophylaxis was sufficiently large to support a positive but anticipated smaller benefit in a future randomized trial. Additionally, in our study most patients were treated with a PEG‐ASP dose of 2500 IU/m^2^ regardless of age, and COG studies have only recently begun to incorporate lower dosing in older patients; this may have affected the rates of hepatotoxicity in our study, and differences between hepatotoxicity rates with different PEG‐ASP dosing should be evaluated further in future studies.

Concurrent medication data was not collected in the rescue group, and synergy between levocarnitine and other vitamin complexes could not be assessed in this study. It will be crucial to collect this information in any forthcoming prospective trial of levocarnitine hepatoprotection. Finally, while raw laboratory data was collected, it is important to note that serial laboratory screening was not performed as it would be in the context of a prospective trial. Despite these limitations, given that levocarnitine was well‐tolerated, with no evidence for chemotherapy interactions *in vitro* ^39^ or influence on disease response in this study, our findings do not controvert current levocarnitine usage or recommendations as an intervention with limited risk and potential hepatic protection. Patients who are older or obese constitute an at‐risk population warranting hepatoprotection. Thus, validating effective strategies to reduce hepatotoxicity is essential to improve the safe delivery of asparaginase‐containing treatment regimens to AYA patients.

## CONFLICT OF INTEREST

Advisory board, Jazz Pharmaceuticals (E.O., J.M.); Speaker's bureau, Servier Pharmaceuticals (V.H.)

## Supporting information

Supplementary Material

## Data Availability

The data that support the findings of this study are available on request from the corresponding author. The data are not publicly available due to privacy or ethical restrictions.
