# Supplementary material for: Levocarnitine for pegaspargase‐induced hepatotoxicity in older children and young adults with acute lymphoblastic leukemia
Source: Cancer Med. 2021 Sep 16;10(21):7551–60. doi: 10.1002/cam4.4281 (PMC8559504; doi:10.1002/cam4.4281)
Supplement: Supplementary file 1 — Supplementary Material [file CAM4-10-7551-s001.docx]

**Supplemental Data**

**Supplemental Tables**

Supplemental Table 1: Summary of induction regimens

Supplemental Table 2: Multivariable analysis of levocarnitine supplementation started any time prior to onset of hepatotoxicity

Supplemental Table 3: Multivariable analysis of levocarnitine prophylaxis and CTCAE Grade ≥3 Hepatotoxicity

Supplemental Table 4: Multivariable analysis of hyperbilirubinemia and levocarnitine supplementation and survival

**Supplemental Figures:**

Supplemental Figure 1: Event-free and Overall survival for patients with and without dose-liming hyperbilirubinemia

| **Supplemental Table 1: Summary of induction regimens** | |
| --- | --- |
| AALL08P1 | - Vincristine 1.5 mg/m^2^ (max 2 mg) days 1, 8, 15, 22 - Daunorubicin 25 mg/m^2^ days 1, 8, 15, 22 - PEG-asparaginase 2500 IU/m^2^ day 4 - Prednisone 30 mg/m^2^/dose BID days 1-28 - Intrathecal cytarabine day 0 or 1 - Intrathecal methotrexate days 8 and 29 (and also days 15 and 22 if CNS3 status) |
| AALL0232 | - Vincristine 1.5 mg/m^2^ (max 2 mg) days 1, 8, 15, 22 - Daunorubicin 25 mg/m^2^ days 1, 8, 15, 22 - PEG-asparaginase 2500 IU/m^2^ day 4 or 5 or 6 (one dose) - Dexamethasone 5 mg/m^2^/dose BID days 1-14 OR prednisone 30 mg/m^2^/dose BID days 1-28 (randomization) - Intrathecal cytarabine day 0 or 1 - Intrathecal methotrexate days 8 and 29 (and also days 15 and 22 if CNS3 status) |
| AALL1131 | - Vincristine 1.5 mg/m^2^ (max 2 mg) days 1, 8, 15, 22 - Daunorubicin 25 mg/m^2^ days 1, 8, 15, 22 - PEG-asparaginase 2500 IU/m^2^ day 4 - Dexamethasone 5 mg/m^2^/dose BID days 1-14 for patients <10 years old OR prednisone 30 mg/m^2^/dose BID days 1-28 for patients ≥10 years old - Intrathecal cytarabine day 0 or 1, with additional doses for CNS2 patients - Intrathecal methotrexate days 8 and 29 (and also days 15 and 22 if CNS3 status) |
| AALL0434 | - Vincristine 1.5 mg/m^2^ (max 2 mg) days 1, 8, 15, 22 - Daunorubicin 25 mg/m^2^ days 1, 8, 15, 22 - PEG-asparaginase 2500 IU/m^2^ day 4 or 5 or 6 (one dose) - Prednisone 30 mg/m^2^/dose BID days 1-28 - Intrathecal cytarabine day 0 or 1 - Intrathecal methotrexate days 8 and 29 (and also days 15 and 22 if CNS3 status) |
| AALL1231 | - Vincristine 1.5 mg/m^2^ (max 2 mg) days 1, 8, 15, 22 - Daunorubicin 25 mg/m^2^ days 1, 8, 15, 22 - PEG-asparaginase 2500 IU/m^2^ days 4 and 18 (two doses) - Dexamethasone 3 mg/m^2^/dose BID days 1-28 - Intrathecal cytarabine day 0 or 1 - Intrathecal methotrexate days 8 and 29 (and also days 15 and 22 if CNS3 status) |

| **Supplemental Table 2: Multivariable analysis of levocarnitine supplementation started any time prior to onset of hepatotoxicity** | | | |
| --- | --- | --- | --- |
| **Covariable** | **Conjugated bilirubin >3mg/dl.** | | |
|  | **OR** | **95% CI** | **p-value** |
| Age, years |  |  |  |
| <15 years | reference | | |
| ≥15 years | 4.88 | 1.41-16.95 | 0.012 |
| BMI Category^1^ |  |  |  |
| Not obese | reference | | |
| Obese | 5.62 | 1.80-17.51 | 0.003 |
| Ethnicity^2^ |  |  |  |
| Not Hispanic/Latinx | reference | | |
| Hispanic/Latinx | 0.82 | 0.26-2.60 | 0.729 |
| PEG-ASP, # doses |  |  |  |
| One | reference | | |
| Two | 6.16 | 0.89-42.53 | 0.065 |
| Levocarnitine suppl. |  |  |  |
| No levocarnitine | reference | | |
| Prophylaxis | 0.24 | 0.06-0.96 | 0.044 |
| ^1^ Obesity defined according to age/sex adjusted norms for BMI in patients <20 years old (BMI percentile <95% vs ≥95%) and BMI <30 vs ≥30 in patients ≥20 years old). ^2^Restricted to those with known ethnicity. BMI = body mass index, PEG-ASP = pegylated asparaginase. | | | |

| **Supplemental Table 3: Multivariable analysis of levocarnitine prophylaxis and CTCAE Grade ≥3 Hepatotoxicity** | | | |
| --- | --- | --- | --- |
| **Covariable** | **Grade ≥3 Hepatotoxicity** | | |
|  | **OR** | **95% CI** | **p-value** |
| Age, years |  |  |  |
| <15 years | reference | | |
| ≥15 years | 1.66 | 0.75-3.68 | 0.210 |
| BMI Category^1^ |  |  |  |
| Not obese | reference | | |
| Obese | 3.66 | 1.60-8.35 | 0.002 |
| Ethnicity^2^ |  |  |  |
| Not Hispanic/Latinx | reference | | |
| Hispanic/Latinx | 0.99 | 0.41-2.40 | 0.983 |
| PEG-ASP exposure, # doses |  |  |  |
| One | reference | | |
| Two | 1.58 | 0.28-8.99 | 0.605 |
| Levocarnitine suppl. |  |  |  |
| No levocarnitine | reference | | |
| Prophylaxis | 1.14 | 0.38-3.47 | 0.812 |
| ^1^ Obesity defined according to age/sex adjusted norms for BMI in patients <20 years old (BMI percentile <95% vs ≥95%) and BMI <30 vs ≥30 in patients ≥20 years old). ^2^Restricted to those with known ethnicity. BMI = body mass index, PEG-ASP = pegylated asparaginase. CTCAE – Common Terminology Criteria for Adverse Events. | | | |

| **Supplemental Table 4: Multivariable analysis of hyperbilirubinemia and levocarnitine supplementation and survival** | | | | | | |
| --- | --- | --- | --- | --- | --- | --- |
| **Covariables** | **Event-free Survival** | | | **Overall Survival** | | |
|  | **HR** | **95% CI** | **p-value^1^** | **HR** | **95% CI** | **p-value^1^** |
| Age, years | 1.03 | 0.92-1.15 | 0.618 | 1.05 | 0.92-1.20 | 0.476 |
| Presenting WBC, 10K/uL | 1.03 | 1.01-1.05 | 0.010 | 1.03 | 1.00-1.06 | 0.023 |
| BMI Category^2^ |  | | |  |  |  |
| Not obese | reference | | |  | reference |  |
| Obese | 2.29 | 1.04-5.07 | 0.041 | 2.86 | 1.03-7.97 | 0.044 |
| FISH/cytogenetics^3^ |  |  |  |  |  |  |
| Neutral | reference | | |  | reference |  |
| Favorable | § |  |  | § |  |  |
| Adverse | 1.87 | 0.68-5.12 | 0.226 | 1.07 | 0.33-3.50 | 0.909 |
| Unknown | 1.66 | 0.57-4.87 | 0.354 | 0.62 | 0.15-2.64 | 0.521 |
| Dose-limiting c.bili |  |  |  |  |  |  |
| ≤3 mg/dl | reference | | |  | reference |  |
| >3 mg/dl | 3.38 | 1.61-7.06 | 0.001 | 4.75 | 1.90-11.88 | 0.001 |
| Levocarnitine suppl.^1^ | 1.22 | 0.41-3.58 | 0.722 | 0.91 | 0.27-3.13 | 0.886 |
| ^§^No patient with favorable cytogenetics developed an event (n=10). ^1^Multivariable Cox model constructed from prognostic factors, levocarnitine supplementation then tested against model, see methods. ^2^Obesity defined according to age/sex adjusted norms for BMI in patients <20 years old (BMI percentile <95% vs ≥95%) and BMI <30 vs ≥30 in patients ≥20 years old).^2^Classified as per Children’s Oncology Group biology protocol AALL08B1 (modified to include Ph-like signatures as adverse prognostic markers). BMI = body mass index, PEG-ASP = pegylated asparaginase. | | | | | | |

**Supplemental Figure 1: Event-free and Overall survival for patients with and without dose-liming hyperbilirubinemia**

Kaplan-Meier curves for event-free survival (A) and overall survival (B), stratified by presence or absence of conjugated bilirubin >3 mg/dl (c.bili) during induction therapy for B-ALL. Significance assessed by log-rank test.


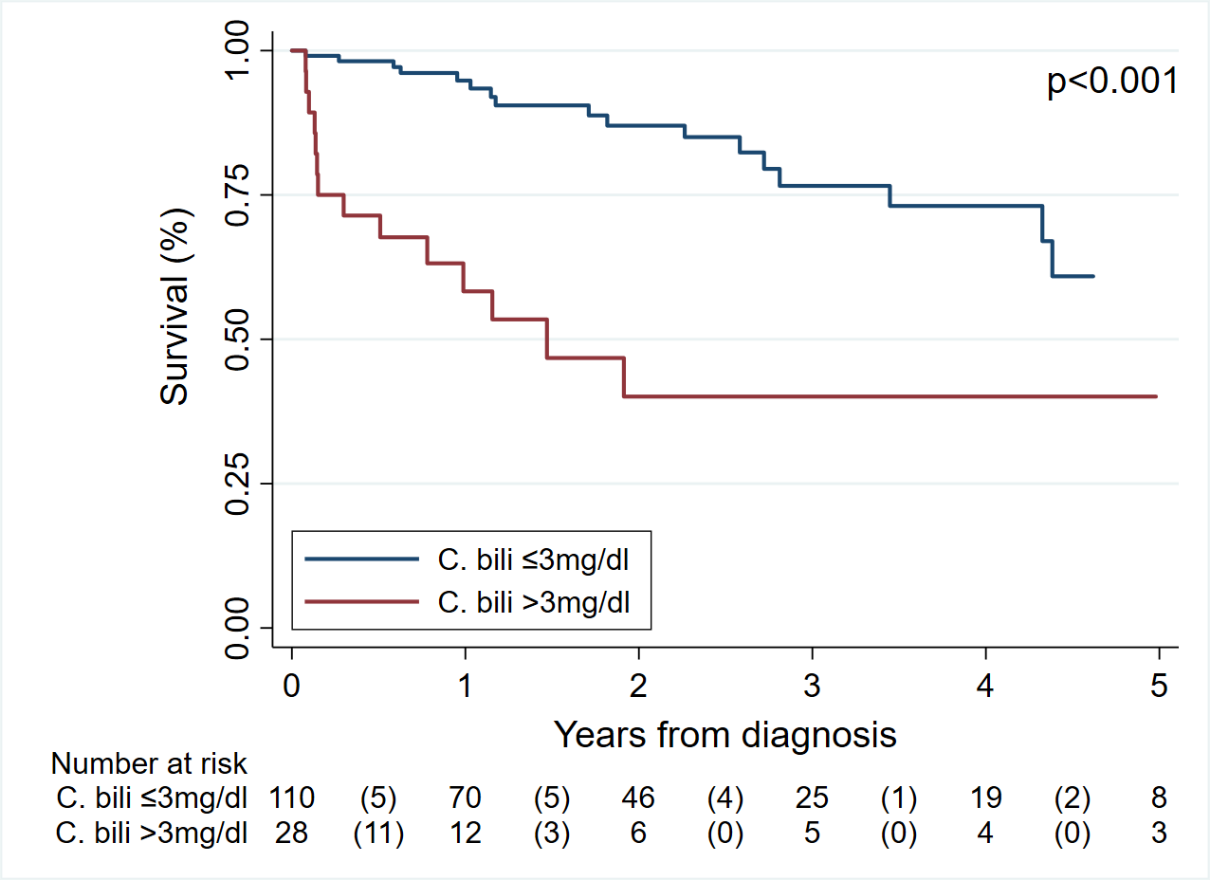


A


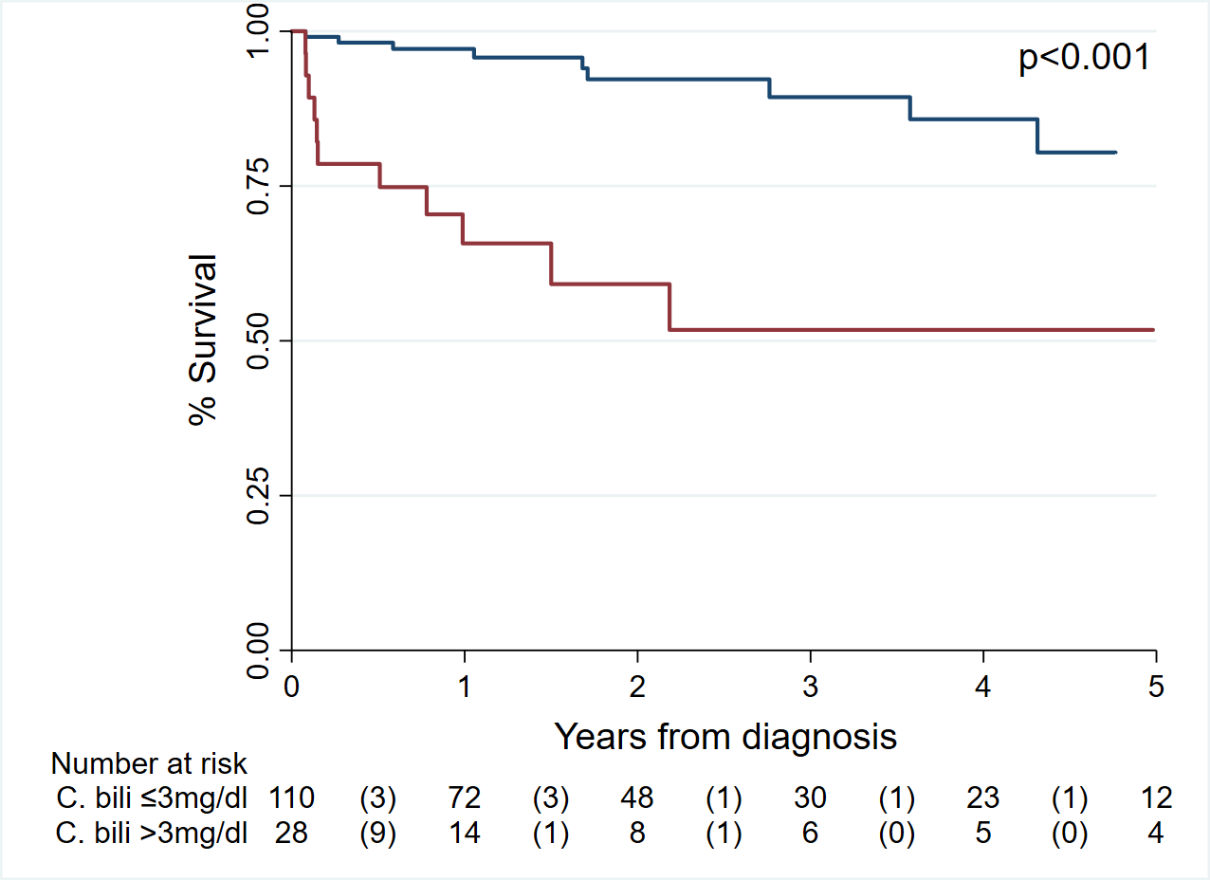


B
